# Prebiotic-Engineered Oral Nanoplatform against Ulcerative Colitis via Photodynamic Remodeling of Gut Microbiota and Macrophage Polarization

**DOI:** 10.34133/bmr.0283

**Published:** 2025-11-21

**Authors:** Ningning He, Huimei Jiang, Tong Dai, Geun-soo Kim, Peng Liu, Yifan Zhao, Shangyong Li, Jie Cao, Zequn Li

**Affiliations:** ^1^ Department of Gastrointestinal Surgery, The Affiliated Hospital of Qingdao University, Qingdao 266000, China.; ^2^ Department of Basic Medicine, Qingdao Medical College, Qingdao University, Qingdao 266071, China.; ^3^ Department of Pharmaceutics, School of Pharmacy, Qingdao University, Qingdao 266071, China.; ^4^ Department of Cell and Developmental Biology, Vanderbilt University, Nashville, TN, USA.

## Abstract

Ulcerative colitis (UC), a chronic inflammatory bowel disease characterized by recurrent colonic mucosal inflammation, substantially impairs patient quality of life. While photodynamic therapy offers promise for UC treatment, conventional photosensitizers face limitations including poor solubility and inadequate targeting. Here, we developed an orally administered multifunctional nanosystem (CBF@LCP) to remodel dysbiotic gut microbiota and enable synergistic phototherapy. The core comprises reactive-oxygen-species-responsive liposomes, encapsulating our previously established iodinated cyanine photosensitizer CyI, and folic acid with bovine serum albumin via amide bonds (CBF@L). This outer layer is coated with a prebiotic chitosan/pectin shell via layer-by-layer assembly. Following oral administration, CBF@LCP withstands the gastrointestinal tract via pH-dependent contraction. Following gastrointestinal-enzyme-mediated decoating, the exposed CBF@L is internalized by folate-receptor-overexpressing M1 macrophages at colitis sites. Under near-infrared irradiation, CyI executes dual photodynamic therapy/photothermal therapy, ablating pro-inflammatory macrophages while exploiting the oxygen-augmented UC microenvironment to enhance reactive oxygen species generation without exogenous oxygen carriers. Concurrently, the prebiotic shell restores microbial eubiosis by suppressing pathogens and promoting beneficial bacteria. In vivo studies in dextran sulfate sodium-induced colitis models demonstrate that CBF@LCP achieves targeted drug release, mitigates inflammation, reprograms macrophage polarization, preserves intestinal barrier integrity, and activates the phosphatidylinositol 3-kinase/AKT signaling pathway. Gut microbiota and transcriptomic analyses confirm restoration of microbial balance and mucosal healing. This work presents a potent targeted strategy for UC management through microbiota remodeling and oxygen-enhanced phototherapy.

## Introduction

Ulcerative colitis (UC) is a chronic inflammatory bowel disease characterized by recurrent inflammation of the colonic mucosa, which can lead to a significant incidence rate and a decline in the quality of life of patients [1]. The pathogenesis of UC is multifactorial, involving genetics, environment, mucosal barrier dysfunction, and aberrant immunity. Current standard therapies, including corticosteroids and immunosuppressants, exhibit limited efficacy and are accompanied by adverse side effects [2]. Therefore, there is an urgent need for innovative treatment strategies that can effectively manage this debilitating condition. Phototherapy, which typically includes photodynamic therapy (PDT) and photothermal therapy (PTT), is recognized as one of the most promising core medical technologies in the field of laser medicine [3]. PDT induces targeted cell apoptosis in inflamed tissues by leveraging photosensitizers to generate reactive oxygen species (ROS) upon light irradiation [4,5]. PTT utilizes photothermal reagents to convert light energy into thermal energy, thereby causing irreversible thermal damage to surrounding cells and tissues [6]. Preclinical studies have demonstrated the safety and efficacy of PDT in the treatment of various gastrointestinal diseases, including UC, reporting a reduction in inflammation and improvement in histopathological parameters [7,8]. In addition, compared with traditional therapies, phototherapy has many advantages as it can provide targeted therapy, minimize systemic side effects, and promote mucosal healing [9–11]. The gut microbiota plays a crucial role in both the pathogenesis of UC and the therapeutic responses to treatment [12]. In a healthy colon, intestinal epithelial cells consume oxygen through β-oxidation, sustaining a strictly anaerobic environment within the lumen. This is further reinforced by anaerobic bacteria, which constitute over 90% of the gut microbiota and maintain a low oxygen partial pressure [13–15]. During the progression of UC, disruption of the mucosal vascular architecture results in microhemorrhage, triggering a substantial accumulation of local red blood cells. Hemoglobin within red blood cells binds and transports oxygen, thereby creating a localized “aerobic microenvironment” at the lesion area [16,17]. Consequently, anaerobic bacteria decline, while pathogenic bacteria expand, activating macrophages and releasing pro-inflammatory factors that perpetuate inflammation. Notably, oxygen-dependent PDT could competitively deplete oxygen with pathogenic bacteria, thereby enhancing ROS yield and eliminating the need for additional oxygen-generating agents that could inadvertently cause cellular damage and promote blood coagulation. However, PDT fails to sufficiently remodel the microbial environment for pathogen suppression, and most photosensitizers suffer from poor aqueous solubility and inadequate targeting specificity. Thus, efficient and targeted delivery of photosensitizers to inflamed colonic sites is essential to optimize therapeutic efficacy and minimize systemic toxicity.

Oral administration is widely preferred for its high patient adherence and favorable safety profile, enabling direct access to inflamed colonic tissues. This approach facilitates both systemic distribution of photosensitizers through intestinal absorption pathways and localized enrichment at inflammatory sites via UC-specific pathological features, such as enhanced vascular permeability and mucosal disruption [18]. However, conventional oral drug delivery systems face multiple barriers in the gastrointestinal tract (GIT). The complex gastrointestinal environment compromises precise drug targeting of inflammatory lesions, causing low local bioavailability and insufficient drug concentrations. These deficiencies not only reduce efficacy but also increase systemic exposure, triggering adverse effects from mild symptoms such as nausea and vomiting to severe complications including pancreatitis [19], hepatotoxicity [20], cardiotoxicity [21], and malignancies [22]. Furthermore, the lack of targeted delivery accelerates drug resistance, necessitating higher doses or more frequent administration and further exacerbating systemic toxicity. Collectively, these limitations demand advanced strategies that maximize local drug accumulation while minimizing off-target effects.

**Fig. 1.**
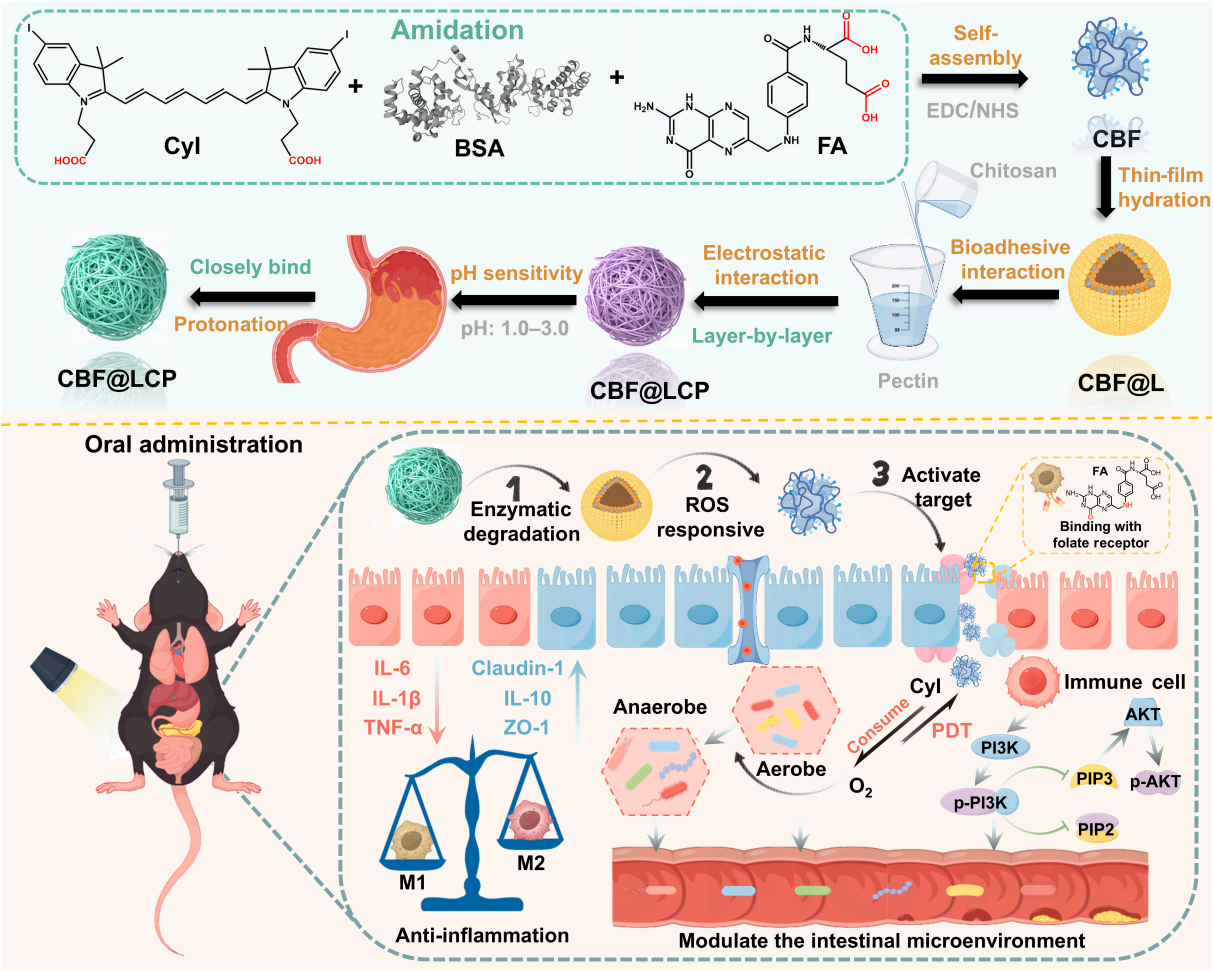
Schematic illustration of CBF@LCP for targeting and phototherapy of ulcerative colitis (UC). CBF@LCP was prepared by layer-by-layer (LBL) assembly and encapsulated the phototherapeutic agent CBF@L. After oral administration, CBF@L is exposed following the action of gastrointestinal enzymes and internalized by folate receptor (FR)-overexpressing M1 macrophages at colitis sites. Reactive oxygen species (ROS)-triggered activation enables CyI-mediated photodynamic therapy (PDT)/photothermal therapy (PTT) ablation of pro-inflammatory M1 macrophages upon near-infrared (NIR) irradiation. The oxygen-augmented environment at the colitis sites can enhance the cell-killing effect of PDT, thereby reversing the microbial homeostasis synergized with pectin/chitosan. Furthermore, immune cell homeostasis can be regulated by phosphatidylinositol 3-kinase (PI3K)–protein kinase B (AKT) signaling to alleviate inflammation in the colitis site. BSA, bovine serum albumin; FA, folic acid; EDC, 1-(3-dimethylaminopropyl)-3-ethylcarbodiimide hydrochloride; NHS, *N*-hydroxy succinimide; CBF, CyI–BSA–FA; IL, interleukin; TNF-α, tumor necrosis factor-α; ZO-1, zonula occludens-1; PIP2, phosphatidylinositol 4,5-bisphosphate; PIP3, phosphatidylinositol (3,4,5)-trisphosphate.

Current photodynamic and photothermal (PDT/PTT) therapies for UC predominantly utilize agents such as 5,10,15,20-tetra{4-[(*S*)-2,6-diaminohexanamido]phenyl}porphyrin (LD_4_) [23,24], 5-aminolevulinic acid [3,8,25], and Foslip [26], which have laid a foundational framework for light-based interventions. However, these approaches are hampered by intrinsic limitations—including simplistic drug delivery designs, limited biofunctionalization of photosensitizers, poor stability in gastric environments, inadequate tissue-specific targeting, and risks of systemic toxicity. In contrast, our study introduces a multifunctional oral nanosystem (CBF@LCP) engineered to overcome these challenges through several strategic innovations (Fig. 1).

The system is constructed using disulfide-containing phospholipids to form ROS-responsive liposomal cores (CBF@L), enabling targeted drug release via cleavage in the high-ROS microenvironment of colitic inflammation. A prebiotic coating—composed of chitosan (Csn) and pectin (Pcn) through layer-by-layer (LBL) self-assembly—enhances biocompatibility, protects the payload throughout gastrointestinal transit, and contracts under acidic conditions, which is induced by the strong electrostatic interaction between the carboxyl group of Pcn and the amino group of Csn, to ensure stability in the stomach. Furthermore, folate-mediated targeting is achieved by co-conjugating the photosensitizer CyI—an iodinated cyanine derivative with a high singlet oxygen quantum yield (ΦΔ = 75%) [27] serving as a dual near-infrared (NIR)-triggered PDT/PTT agent [28]—along with folic acid (FA) to bovine serum albumin (BSA), thereby promoting selective uptake by M1 macrophages that overexpress folate receptors (FRs) and play a critical role in UC pathogenesis.

Upon oral administration and enzymatic decoating in the gut, the exposed CBF@L is internalized by M1 macrophages. ROS-triggered drug release activates CyI-mediated phototherapy under NIR irradiation, effectively ablating pro-inflammatory macrophages. The oxygen-augmented colitic microenvironment potentiates PDT efficacy, synergizing with Pcn/Csn to restore microbial eubiosis through selective pathogen inhibition and beneficial bacteria promotion. Furthermore, immune cell homeostasis can be regulated by phosphatidylinositol 3-kinase (PI3K)–protein kinase B (AKT) signaling to alleviate inflammation in the colitis site.

Beyond these functional improvements, our study provides mechanistic evidence at the molecular level through integrated multi-omics analyses, including transcriptomic profiling and 16S ribosomal RNA sequencing, yielding a more in-depth mechanistic understanding. Thus, the CBF@LCP nanosystem not only addresses critical gaps in existing PDT/PTT-based UC therapies but also provides a more targeted, stable, and mechanistically grounded strategy for effective UC treatment.

## Materials and Methods

### Materials

CyI (Mw 776.5 Da) was synthesized according to our previously reported protocol [29]. FA, dimethyl sulfoxide, and Csn with a deacetylation degree of ≥95.0% and a viscosity range of 100 to 200 mPa·s were purchased from Aladdin (Shanghai, China). Pcn with a galactose uronic acid content of ≥74%, BSA, and phosphate-buffered saline (PBS; 0.1 mol/l, pH = 5) were obtained from Yuanye Bio-Technology Co., Ltd. (Shanghai, China). Lecithin, cholesterol, *N*-hydroxy succinimide (NHS), and 1-(3-dimethylaminopropyl)-3-ethylcarbodiimide hydrochloride (EDC) were purchased from Macklin (Shanghai, China). DSPE-PEG_2000_ was purchased from Ruixi Biotech Co., Ltd (Xian, China). 2′,7′-Dichlorodihydrofluorescein diacetate (DCFH-DA) and singlet oxygen sensor green (SOSG) were bought from Dalian Meilun Biotechnology Co., Ltd. (Dalian, China). 4′,6-Diamidino-2-phenylindole (DAPI) solution was purchased from Solarbio (Beijing, China). Antibodies for AKT (no. 9272), phospho-AKT (no. 9271), PI3K p85 (no. 4292), and phospho-PI3 kinase p85 (no. 4228) were from CST Corporation (Boston, USA). Antibodies against zonula occludens-1 (ZO-1; GB151981), claudin-1 (GB12032), occludin (GB111401), CD206 (GB113497), and inducible nitric oxide synthase (iNOS; GB11119) were purchased from Servicebio (Wuhan, China). RAW 264.7 and Caco-2 were obtained from Wuhan Procell Life Science & Technology (Wuhan, China). Cells were cultured in Dulbecco’s modified Eagle medium (DMEM; Procell, USA) and minimum essential medium (Procell, USA) with 10% fetal bovine serum (Procell, USA) and 1% penicillin–streptomycin (HyClone, USA), respectively. C57BL/6J mice (6 weeks, male, 18 to 22 g) were obtained from Pengyue Experimental Animal Breeding Co., LTD (Jinan, China).

### Preparation of CBF@LCP

FA and CyI were activated in dimethyl sulfoxide solution of EDC and NHS, respectively, for 4 h, and then added drop by drop into a PBS solution of BSA, adjusting the mass ratio to 4:2:1, and the reaction was protected from light for 12 h. PBS gradient dialysis was performed for 3 d using dialysis bags (molecular weight cutoff: 8,000 to 12,000) to remove excess raw materials and by-products. Finally, the final product was obtained by freezing the solution in the dialysis bag.

The morphological characteristics of BSA and CyI–BSA–FA (CBF) were detected by transmission electron microscopy (TEM; JEM2010, JEOL, Japan). The hydration particle size and zeta potential of CBF were determined using a Mastersizer Nano-ZS90 laser particle size analyzer (Malvern, UK). The standard curves of CyI and FA were plotted with ultraviolet–visible–near-infrared (UV–vis–NIR) spectroscopy, and the amounts of CyI and FA in CBF were determined at wavelengths of 780 and 360 nm. The UV–vis absorbance spectra of BSA, FA, CBF, and CyI were acquired using UV–vis–NIR spectroscopy. The fluorescence spectra of CyI and CBF were obtained using a fluorescence spectrophotometer (Ex = 810 nm). Fourier transform infrared (FTIR) spectroscopy was used to analyze the samples (BSA, FA, CBF, and CyI) with an FTIR spectrometer in the range of 4,000 to 400 cm^−1^. Before the measurement, the samples were dried under vacuum until a constant weight was reached. The dried samples were pressed into the powder, mixed with KBr powder, and then pressed into a shape for FTIR spectroscopy to measure the wavelength.

The liposomes were prepared by using the thin-film hydration method. In brief, in eggplant bottles, lecithin, cholesterol, and DSPE-PEG2000 were dissolved in chloroform at a molar ratio of 5:1:1. The organic solvent was evaporated under reduced pressure. A solution of CBF (3 mg) in PBS (5 ml) was prepared. Using an ultrasonic molecular breaker, it was sonicated for 2 min at 20 W to obtain a liposome dispersion solution with a uniformly stable particle size. The LBL method was employed to encapsulate CBF@L with Pcn and Csn as the probiotic shell. Csn solution (1% w/v acetic) was slowly added to the CBF@L solution while stirring for 1 h. Pcn solution was then added dropwise into the mixture while stirring for 1 h as well. Subsequently, the final CBF@LCP was obtained after lyophilization.

The formed CBF@L, CBF@LC, and CBF@LCP were observed under TEM. Their hydration particle size and zeta potential were determined by dynamic light scattering (DLS). The amount of CBF in CBF-Liposome was determined at a wavelength of 780 nm. The UV–vis absorbance spectra of CBF and CBF@L were acquired using UV–vis–NIR spectroscopy. The fluorescence spectra of CBF and CBF@L were obtained using a fluorescence spectrophotometer (Ex = 810 nm). The storage stability of CBF@L was evaluated for 48 h at 37 °C and for 6 d at 4 °C.

### In vitro drug release

To simulate the release rate of CBF@LCP in the GIT, 3 different pH levels were employed in vitro to mimic the acidic environment of the stomach (pH 1.2), small intestine (pH 6.8), and colon (pH 7.4) following the methods described in our previous study [30]. To evaluate the enzyme-responsive release behavior of CBF@LCP, a solution containing fecal microbiota suspension at a concentration of 50 mg/ml or pectinase at 125 mg/ml was utilized. The drug release (CBF@LCP) was quantified at a wavelength of 780 nm. The cumulative drug release was calculated using the following formula:

$$\begin{array}{c} \text{Cumulative drug release}\;\%=\\ \frac{\text{Cumulative drug release}\;}{\text{Total amount of particle loading}\;\left(\mathrm{mg}\right)}\times 100\;\left(\%\right) \end{array}$$ (1)

### PDT and PTT properties of CBF@LCP

One hundred fifty microliters of the samples (PBS, CyI, CBF, CBF-Liposome, and CBF@LCP) was taken in a 96-well plate, and 50 μl of SOSG was added and mixed well. We irradiated the samples with an 808-nm NIR laser (0.96 W/cm^2^) for different durations. The fluorescence intensity of each set of samples was recorded with FlexStation (SOSG Ex = 504 nm and Em = 525 nm), and the measurement was repeated 3 times in parallel. The temperature changes of different samples (PBS, CyI, CBF, CBF-Liposome, and CBF@LCP) under an 808-nm NIR laser at 0.96 W/cm^2^ irradiation were recorded using a thermocouple thermometer and an infrared thermal imaging camera.

### In vitro targeting of M1 macrophages by CBF

To evaluate the targeting properties, RAW 264.7 cells were firstly seeded onto 35-mm^2^ confocal dishes at a density of 2 × 10^5^ cells/ml and then 1 μg/ml lipopolysaccharide (M1-like) or interleukin-4 (IL-4; M2-like) was added to stimulate the polarization of RAW 264.7 cells to the M1 or M2 phenotype. Uninduced RAW 264.7 cells were M0 macrophages. After the induction was completed, CBF (FA: 86 μg/ml; CyI: 50 μg/ml) was added and incubated for 6 h and photographed by STELLARIS 5 Confocal Microscope (STELLARIS 5, Leica, Germany). In addition, we performed blocking experiments to explore the mechanism by which CBF targets M1 macrophages. Simply, the M1 macrophages were pretreated with 1 mM free FA for 1 h to block FR before incubating with CBF (80 μg/ml). Unblocked treated M1 macrophages were incubated for 6 h with the addition of CyI–BSA (CB) and CBF, respectively. All of the above treated cells on confocal dishes were fixed with 4% paraformaldehyde for 10 min and stained with DAPI for 10 min, followed by observing using a confocal laser scanning microscope.

The cellular uptake of CBF@LCP was studied at different times with a confocal laser scanning microscope. Briefly, M1 macrophages were seeded onto 35-mm^2^ confocal dishes at a density of 1 × 10^5^ cells/ml. Then, the medium was sucked out and treated with CBF@LCP (80 μg/ml) for 2, 4, 6, and 8 h, respectively, followed by washing 3 times with PBS (pH = 7.4). The cells were fixed with 4 % paraformaldehyde for 10 min and stained with DAPI (2 μg/ml) for 10 min. Then, after washing the cells 3 times with PBS, images were acquired by a confocal laser scanning microscope. Furthermore, the cell uptake was measured by a flow cytometer (CytoFLEX S, Beckman Coulter, China).

### In vitro targeting of CBF to M1 macrophages

To evaluate the targeting properties, RAW 264.7 cells were firstly seeded onto 35-mm^2^ confocal dishes at a density of 2 × 10^5^ cells/ml and then 1 μg/ml lipopolysaccharide (M1-like) or IL-4 (M2-like) was added to stimulate the polarization of RAW 264.7 cells to the M1 or M2 phenotype. Uninduced RAW 264.7 cells were M0 macrophages. After the induction was completed, CBF (FA: 86 μg/ml; CyI: 50 μg/ml) was added and incubated for 6 h and photographed by STELLARIS 5 Confocal Microscope (STELLARIS 5, Leica, Germany). In addition, we performed blocking experiments to explore the mechanism by which CBF targets M1 macrophages. Simply, the M1 macrophages were pretreated with 1 mM free FA for 1 h to block FR before incubating with CBF (80 μg/ml). Unblocked treated M1 macrophages were incubated for 6 h with the addition of CB and CBF, respectively. All of the above treated cells on confocal dishes were fixed with 4% paraformaldehyde for 10 min and stained with DAPI for 10 min, followed by observation using a confocal laser scanning microscope.

The cellular uptake of CBF@LCP was studied at different times with a confocal laser scanning microscope. Briefly, M1 macrophages were seeded onto 35-mm^2^ confocal dishes at a density of 1 × 10^5^ cells/ml. Then, the medium was sucked out and treated with CBF@LCP (80 μg/ml) for 2, 4, 6, and 8 h, respectively, followed by washing 3 times with PBS (pH = 7.4). The cells were fixed with 4 % paraformaldehyde for 10 min and stained with DAPI (2 μg/ml) for 10 min. Then, after washing the cells 3 times with PBS, images were acquired by a confocal laser scanning microscope. Moreover, the cell uptake was measured by a flow cytometer (CytoFLEX S, Beckman Coulter, China).

### Phototherapy property of CBF-Liposome within cells

In order to validate the photodynamic effect of CBF@LCP, a DCFH-DA probe was used to detect the production of singlet oxygen. In brief, M1 macrophages were inoculated into small dishes co-incubated with different drugs (control, NIR, BSA, CBF, CBF-Liposome, and CBF@LCP) for 6 h. Next, the cell culture medium was removed, and diluted DCFH-DA probes were added to each group to incubate for 20 min in the cell incubator. Then, cells were irradiated with an 808-nm laser for 5 min and further incubated in an incubator for 20 min. Finally, the cells were collected and analyzed by a laser confocal microscope and flow cytometry. The performance of PTT was measured by a thermocouple thermometer and an infrared thermal imaging camera. The M1 macrophages were incubated with different solutions for 6 h. Next, the cells were collected and resuspended in PBS. All groups were irradiated for 5 min. The final temperature of every group was recorded by an infrared thermal imaging camera.

### Cell cytotoxicity test

Caco-2 and RAW 264.7 cells were cultured in an incubator at 37 °C with 5% CO_2_. After cell counting, 3,000 cells were seeded into each well of 96-well plates. Various concentrations of CBF@LCP were then added, and the cultures were incubated for 48 h. The cytotoxicity of CBF@LCP was evaluated using the Cell Counting Kit-8 assay. This experimental procedure provides a standardized method for assessing the effect of CBF@LCP on cell viability and is essential for evaluating its potential toxicity.

### Animal experiment

Animal protocols were reviewed and approved by the Ethics Committee of the Medical College of Qingdao University (QDU-AEC-2023388). All animal procedures were conducted in compliance with applicable regulations and the ARRIVE guidelines. Following a 1-week acclimation period, the mice were randomly assigned to 5 groups (*n* = 8) and subjected to the following treatments: (a) NC group: no treatment; (b) dextran sulfate sodium (DSS) group: 2.5% DSS for 1 week; (c) CBF@LCP group: 2.5% DSS for 1 week, with CBF@LCP (5 mg/kg) administered via gavage on the fourth day; (d) NIR group: 2.5% DSS for 1 week, with PBS gavage and NIR irradiation (808 nm, 0.96 W/cm^2^) on the fourth day; and (e) CBF@LCP+NIR group: 2.5% DSS for 1 week, with CBF@LCP (5 mg/kg) administered via gavage and NIR irradiation (808 nm, 0.96 W/cm^2^) on the fourth day. Colitis was induced by providing the mice with 2.5% DSS dissolved in drinking water. The severity of intestinal inflammation was monitored daily by recording the body weight and calculating the disease activity index (DAI), which includes assessments of weight changes, fecal consistency, and the presence of blood in the stool. Following gas anesthesia with 3% isoflurane, blood samples were collected from the orbit. Then, the mice were euthanized by cervical dislocation and the colon was excised for length measurements and histological examination. Colon tissues were collected and stored at −80 °C for further analysis.

### Histological evaluation and measurement of inflammatory cytokines in serum

Histopathological changes in colon tissues were assessed through hematoxylin and eosin (H&E) staining, while mucin expression was analyzed via Alcian Blue staining. The stained tissue sections were examined under an Olympus microscope (Olympus, Tokyo, Japan) at ×40 and ×200 magnification. H&E staining was scored histologically using a blinded approach, and the quantification of Alcian Blue staining was conducted using the ImageJ software.

Blood samples were centrifuged at 3,000 rpm for 15 min to obtain serum. The levels of inflammatory cytokines, including tumor necrosis factor-α (TNF-α), IL-1β, IL-6, and IL-10, were measured using enzyme-linked immunosorbent assay kits (ABclonal, Wuhan, China) following the manufacturer’s instructions.

### Immunohistochemistry analysis

For the purpose of immunohistochemistry analysis, tissue sections embedded in paraffin were subjected to antigen retrieval by boiling in a sodium citrate buffer. The slides were then treated with a 3% hydrogen peroxide solution to block endogenous peroxidase activity, followed by incubation with 3% BSA for 30 min at room temperature to minimize nonspecific binding. After overnight incubation with the primary antibodies (1:500) at 4 °C, the sections were exposed to the secondary antibody. Subsequently, the slides were stained with 3,3′-diaminobenzidine and hematoxylin to visualize the target antigen. Finally, the stained sections were examined under a microscope (Olympus, Tokyo, Japan).

### Quantitative real-time polymerase chain reaction and western blot analysis

Total RNA extraction and reverse transcription were conducted following the protocol provided by the RNA extraction kit (no. AC0202, Sparkjade, Jinan, Shandong, China). Primers, as listed in Table S1, were designed and synthesized by Sangon Biotech (Shanghai, China). The relative gene expression was quantified using the comparative threshold cycle method (2^−ΔΔCT^). For protein extraction, 100 mg of tissue samples was processed with radioimmunoprecipitation assay lysis buffer supplemented with protease and phosphatase inhibitors (Beyotime Biotechnology, Shanghai, China). The protein concentration was determined using a bicinchoninic acid assay kit. Western blot analysis was carried out as previously described [31].

### 16S ribosomal RNA gut microbiota analysis

DNA was isolated from fecal samples using the Stool DNA Kit (Omega Bio-Tek, Norcross, GA, USA) following the manufacturer’s guidelines. The purity and concentration of the extracted DNA were measured with a NanoDrop spectrophotometer, while its quality was assessed through gel electrophoresis. Specific regions of the variable regions (V3 to V4) were amplified using the universal primers 338F (5′-ACTCCTACGGGAGGCAGCAG-3′) and 806R (5′-GGACTACHVGGGTWTCTAAT-3′). The amplified DNA fragments were then purified and sequenced using the NovaSeq 6000 platform (Illumina, San Diego, CA, USA). DNA extraction and sequencing services were provided by Novogene Technology Co., Ltd. (Beijing, China).

### Transcriptome analysis

Total RNA was isolated from the tissue using TRIzol reagent, in accordance with the manufacturer’s protocol. Only RNA samples of high quality were selected for sequencing library construction. RNA purification, reverse transcription, library preparation, and sequencing were performed at Novogene Technology Co., Ltd. (Beijing, China), following the manufacturer’s instructions (Illumina, San Diego, CA, USA). The RNA sequencing transcriptome library was prepared using the Illumina Stranded mRNA (messenger RNA) Prep, Ligation kit from Illumina (San Diego, CA, USA), employing 1 μg of total RNA. The raw paired-end reads were trimmed and subjected to quality control using fastp v0.23.4 with default parameters. Subsequently, the clean reads were aligned separately to the reference genome in orientation mode utilizing the HISAT2 v2.1 software. The mapped reads from each sample were subsequently assembled using StringTie through a reference-based approach. To identify differentially expressed genes (DEGs) between the 2 samples, transcript expression levels were calculated using the transcripts per million reads method. Gene abundances were quantified with RSEM v1.3.3. Differential expression analysis was conducted using DESeq2 v1.44.0.

### Statistical analysis

Statistical analyses were conducted using GraphPad (GraphPad Software, San Diego, CA, USA) and R software. Data are expressed as mean ± standard deviation or mean ± standard error of the mean. Differences between groups were examined using an unpaired Student *t* test, one-way analysis of variance, the Kruskal–Wallis test, or the Mann–Whitney *U* test (for nonnormally distributed data). Spearman correlation analysis was employed to evaluate associations between variables. A *P* value of less than 0.05 was considered statistically significant.

## Results and Discussion

### Preparation and characterization of CBF@LCP

PDT has been demonstrated to be effective in alleviating symptoms of various inflammatory conditions, including arthritis [29,32] and acne [33]. Its application in UC has also shown promising results [23]. Given the unique clinical features of UC, which is characterized by its persistent inflammation of the colonic mucosa and disorder impact on the gut microbiota, we meticulously designed an oral treatment approach that not only targets the inflammatory process but also maintains the delicate balance of the gut microbiota. Oral drug delivery systems offer advantages including the simplicity of administration and minimal side effects, making them particularly suited for colonic-targeted therapies, such as those for UC and colorectal cancer, enhancing treatment efficacy as well as reducing systemic toxicity [34]. To pass through the GIT safely and locate in the colon precisely, it is deficient to utilize a photosensitizer singly as for the existence of the potential risks of cytotoxicity and the lack of ability to defend the invasion of gastric acid and target the colon tissue [27], and that means modification and encapsulation for photosensitizer are needed to make up for its shortness.

To develop a core–shell-structured PDT system suitable for oral administration in the treatment of UC, we utilized a Csn–Pcn shell to encapsulate CyI, thereby constructing an oral nanodrug system with potential for targeted release. To enhance the hydrophilicity of CyI and FA, BSA was employed as a stabilizing agent for the synthesis of the CBF core through chemical interactions between the carboxyl groups of CyI and FA and the amino groups of BSA. To enhance the retention time at the sites of colitis, CBF was encapsulated in liposomes (designated CBF@L). Finally, CBF was encapsulated with a Csn–Pcn shell using the thin-film hydration method and 2 cycles of the LBL technique, forming CBF@LCP nanoparticles (NPs).

**Fig. 2.**
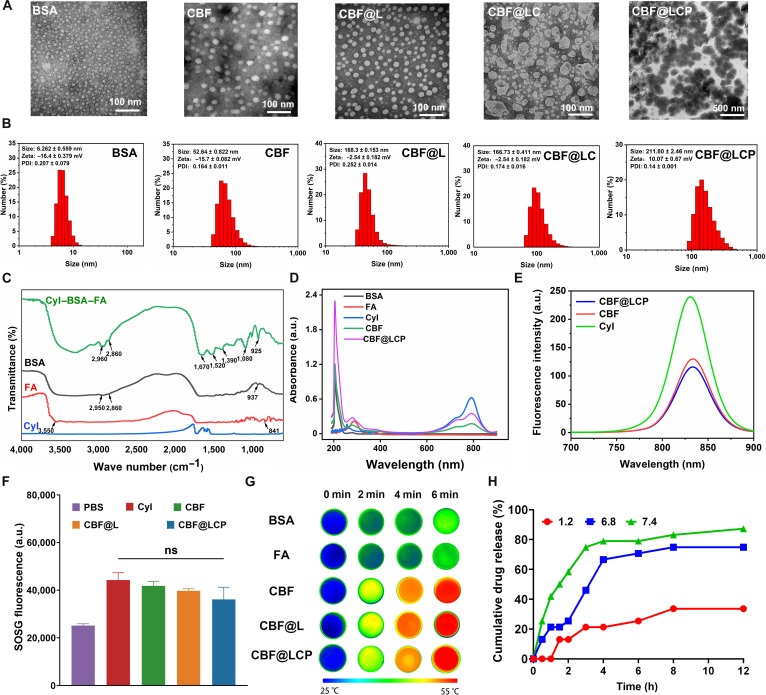
The preparation and characterization of CBF@LCP. (A) Transmission electron microscopy (TEM) images. (B) Hydrodynamic diameter analysis. (C) Fourier transform infrared (FTIR) spectra of CyI, FA, BSA, and CBF@LCP. (D) ultraviolet–visible (UV–vis) spectrum measurement (E) Fluorescence spectra of CyI, CBF, and CBF@LCP (Ex: 810 nm). (F) Singlet oxygen production under NIR irradiation (808 nm, 0.96 W/cm^2^, 5 min) (*n* = 3). (G) Infrared thermography of different samples under varying irradiation times. (H) Release curves of CBF@LCP at different pH conditions. PDI, polydispersity index; SOSG, singlet oxygen sensor green; ns, not significant.

The morphology and size of BSA, CBF, CBF@L, CBF@LC, and CBF@LCP were characterized by TEM and DLS. As shown in Fig. 2A and B, both BSA and CBF were monodispersed protein spheres, and their hydrodynamic diameters were 6.262 ± 0.559 and 52.64 ± 0.822 nm, respectively. Meanwhile, the polydispersity index value of the formed CBF was 0.164 ± 0.011, indicating a narrow particle size distribution. As expected, CBF@L dispersed as individual spherical NPs, with a double-membrane structure and a uniform particle size. After 2 cycles of LBL assembly, the resulting average particle size of CBF@LCP was determined to be 212.03 ± 2.41 nm, which is consistent with the observation in TEM. The zeta potentials of BSA, CBF, CBF@L, CBF@LC, and CBF@LCP were measured to be −16.4 ± 0.379, −15.7 ± 0.082, −2.54 ± 0.182, 17.63 ± 0.34, and 10.07 ± 0.67 mV, respectively. The significant change in CBF@L potential compared to that of CBF may be due to the effect of the liposome outer layer. Note that the size measured by DLS was significantly larger than that measured by TEM, possibly due to shrinkage of BSA, CBF, and CBF@L during TEM sample preparation. Additionally, our final NPs ought to have commendable biosecurity, and related cell viability tests where our CBF@LCP was incubated with Caco-2 and RAW 264.7 cells, respectively, demonstrated no evidence of its potential toxicity, being consistent with our hypothesis (Fig. S1).

To further analyze the functional groups and confirm CyI and FA conjugation, FTIR analysis was carried out for CyI, FA, BSA, and CBF@LCP (Fig. 2C). As expected, the spectrum of BSA exhibited characteristic peaks at 1,600 to 1,500 cm^−1^. Meanwhile, the typical peaks for FA were observed at 3,550 and 841 cm^−1^, corresponding to the N–H bond stretching of primary amine and the absorption band of the phenyl ring, respectively. The interaction between CyI and FA leads to changes in protein conformation, resulting in a spectral shift for the protein amide I band at 1,670 cm^−1^ (mainly C=O stretching) and the amide II band at 1,520 cm^−1^ (C–N stretching coupled with N–H bending modes). The spectrum of CBF@LCP showed peaks corresponding to BSA and FA, indicating successful coupling. The reduction in the intensities of the respective bands is an indication of the amorphous nature of the material with a lower weight percentage of the individual component in the coupling carrier. Based on the standard curve of CyI and FA (Fig. S2), the coupling ratios of CyI and FA were calculated to be 46.04% ± 0.148% and 80.25% ± 0.312%, respectively. As shown in Fig. 2D, characteristic peaks at 220 nm (BSA), 360 nm (FA), and 798 nm (CyI) were clearly observed in the absorption spectra of CBF and CBF@LCP, indicating the successful formation and encapsulation of CBF. The encapsulation efficiency and the loading efficiency of CBF were calculated to be 95.19% ± 0.358% and 0.968% ± 0.454%, respectively. As shown in Fig. 2E, CyI, CBF, and CBF@LCP exhibit an identical fluorescence emission peak at about 810 nm under excitation with 780-nm light, indicating that CBF@LCP, when excited in the NIR region, can be used for fluorescence imaging. Next, the stability of CBF@L in an aqueous medium containing serum, PBS, water, and DMEM at 4 °C was examined by DLS. Results showed that there was no significant particle diameter change (Fig. S3A and B). These results showed that no obvious size changes were observed under different conditions, indicating the good physical stability of CBF@L.

SOSG, a single linear oxygen-specific fluorescent probe that reacts with CyI-generated ^1^O_2_ and produces green fluorescence, was used to determine the ^1^O_2_ production capacity of CBF@LCP. Compared with that of the PBS group, a large amount of ^1^O_2_ was generated in the CyI, CBF, CBF@L, and CBF@LCP groups (Fig. 2F). This indicates that the in vitro PDT effect of CBF is mainly dependent on CyI. Furthermore, it suggests that the modification of CyI and its encapsulation within liposomes do not affect the PDT efficacy of CyI after 5 min of NIR irradiation (808 nm, 0.96 W/cm^2^). Photothermal action (>50 °C) has been extensively utilized for the eradication of malignant tissues by inducing irreversible physical damage. Therefore, we investigated the photothermal conversion efficiency of CBF under NIR irradiation. Following 5 min of NIR irradiation, the temperatures of the BSA and FA groups remained relatively constant, whereas the temperatures of CBF, CBF@L, and CBF@LCP increased by 20 °C within 3 min. These results show that the photothermal action of CyI can lead to temperatures up to 55 °C and that it can also act stably within couplings and liposomes. The photothermal images further confirmed the results (Fig. 2G).

Efficient transit through the GIT is essential for NPs intended for in vivo oral administration. CBF@LCP demonstrated the highest release rate at pH 7.4, while only 33.63% of CBF was released at pH 1.2 after 12 h (Fig. 2H). The strong electrostatic interaction between the carboxyl group of Pcn and the amino group of Csn induces CBF@LCP to contract in low-pH conditions, thereby protecting CBF in the GIT and the acidic environment of the stomach [35]. Furthermore, when CBF@LCP was combined with a bacterial solution (50 mg/ml) or pectinase solution (125 mg/ml), a significant enhancement in the release of CBF was observed within 8 h, achieving approximately 95.46%. These findings indicate that CBF@LCP displays pH sensitivity in the colon and responds to pectinase produced by gut microbiota, thereby showing significant potential for controlled release within colonic tissues.

### In vitro cellular uptake evaluation

**Fig. 3.**
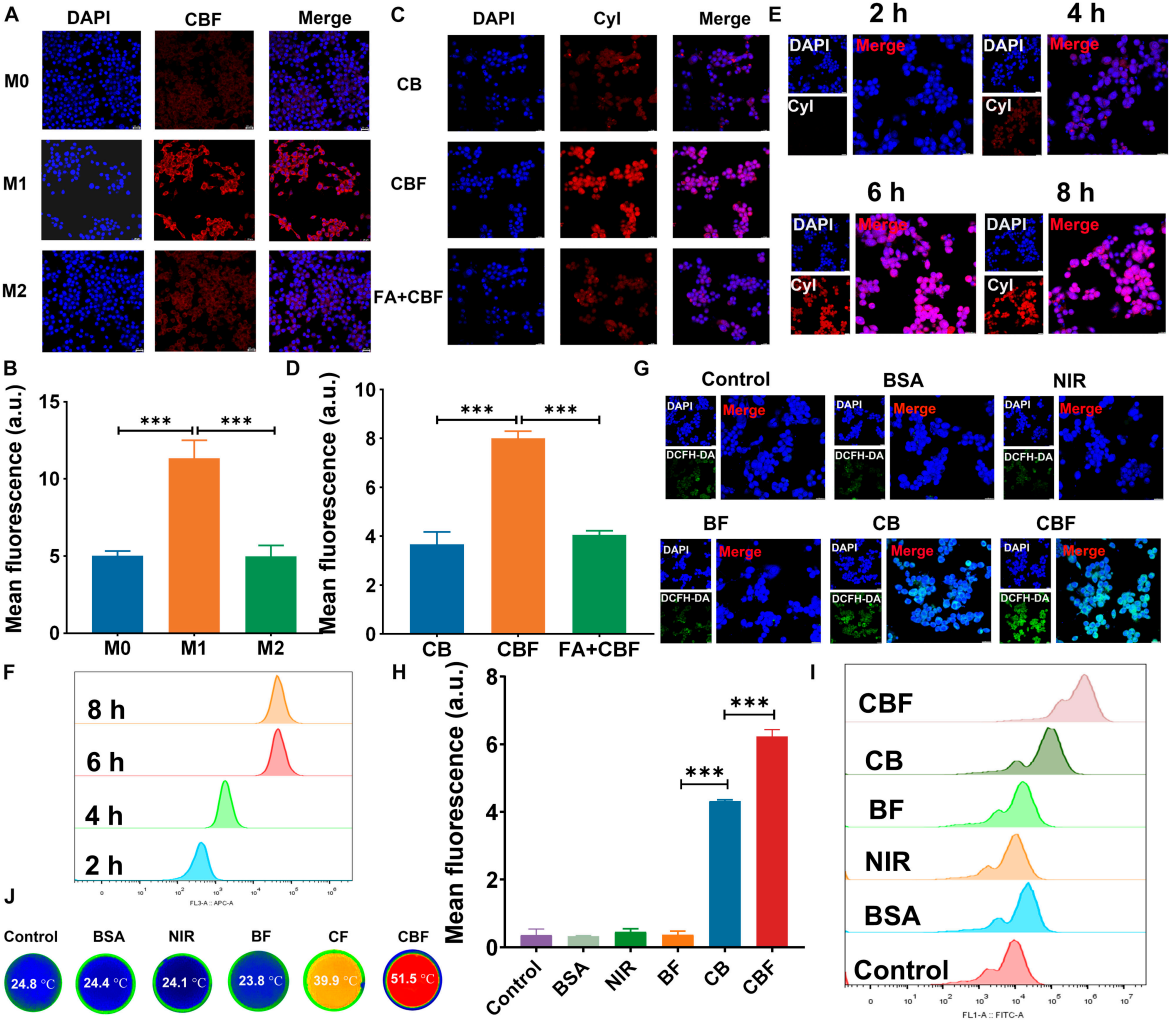
Cellular uptake evaluation and CBF@LCP phototherapy in vitro. (A) Confocal fluorescence images of CBF (CyI: 50 μg/ml) incubated with different types of macrophages. (B) Mean fluorescence intensity. (C) Confocal fluorescence images of M1 macrophages incubated with CyI–BSA (CB), CBF, and FA + CBF for 6 h. (D) Mean fluorescence intensity. (E) Confocal fluorescence images of M1 macrophages cultured with CBF@LCP for 2, 4, 6, and 8 h. (F) Flow cytometry was used to analyze the uptake of M1 macrophages after incubation for different times. (G) Confocal images of intracellular ROS production in M1 macrophages after incubation with different samples. (H) Mean fluorescence intensity of M1 macrophages with different samples. (I) Flow cytometry was used to analyze ROS production after different samples’ cultures of M1 macrophages. (J) Representative thermal images of M1 macrophages under laser irradiation (808 nm, 0.96 W/cm^2^). Scale bar: 20 μm. Data are presented as mean ± SD (*n* = 3). ***P < 0.001. DAPI, 4′,6-diamidino-2-phenylindole; DCFH-DA, 2′,7′-dichlorodihydrofluorescein diacetate.

To verify whether the synthesized FA-conjugated NPs could target M1 macrophages, the synthetic CBF was incubated with various macrophage types (M0, M1, and M2) for an identical duration, and the results were validated using a laser confocal microscope. As shown in Fig. 3A, M1 macrophages preferentially uptake CBF with a significantly higher fluorescence intensity (red) than M0 and M2. Consistent results were observed in the confocal laser scanning microscopy (CLSM) images and semiquantitative analyses (Fig. 3B), likely due to the activation of M1 macrophages by inflammatory stimuli, enhancing their albumin internalization ability substantially through increased surface expression and activity of the FRs on their surface. Furthermore, to further investigate the factors influencing the targeting of M1 macrophages by CBF, we observed that the uptake of CBF by M1 macrophages was significantly higher compared to that of BC. This indicates that the presence of FA on CBF plays a crucial role in enhancing its specificity toward M1 macrophages. To further validate the role of FR in the uptake of CBF by cell surface receptors, we performed FA blocking experiments. As shown in Fig. 3C, free FA resulted in a significant limitation of the macrophage uptake of CBF due to blocking the FR overexpressed on M1 macrophages. The semiquantitative results (Fig. 3D) further verified that the FR-mediated uptake pathway plays an important role in CBF targeting of M1 macrophages. Targeting of M1 macrophages by CBF using FR allows the PDT and PTT effects produced by CyI to kill pro-inflammatory M1 macrophages to a greater extent, alleviating UC inflammation, reducing damage to other cells, and increasing the bioavailability of CBF.

The timing of cellular uptake of CBF by M1 macrophages was evaluated using CLSM and flow cytometry. As shown in Fig. 3E and Fig. S6, M1 macrophages displayed a strong red fluorescent signal after 6 and 8 h of CBF incubation with no significant difference. Next, we verified that the above experimental results were consistent with the CLSM results, which further confirmed that CBF could be effectively taken up by M1 macrophages and the uptake was saturated at 6 h. The results of the flow cytometry were also consistent with the CLSM results.

### In vitro CBF phototherapy

DCFH-DA as an ROS detector becomes fluorescent DCF in the presence of ROS and is often used to determine the generation of ROS. As shown in Fig. 3G, compared to that of the control group, the green fluorescence intensity of the BSA, NIR, and BF groups did not have obvious differences, indicating that neither NIR nor BSA had the ability to produce ROS. In contrast, the green fluorescence signals of CB and CBF increased, demonstrating that the CyI-based nanosystem effectively generated ROS in M1 macrophages (RAW 264.7) after laser irradiation. Notably, the fluorescence intensity of CBF was significantly higher than that of CB, which may be due to the high affinity of CBF for FR on the surface of M1 macrophages, which may increase the uptake of CBF by M1 macrophages, leading to increased ROS. These results were further validated by flow cytometry and semiquantitative analysis (Fig. 3H and I).

Subsequently, we investigated the photothermal effect of CBF in M1 macrophages by infrared thermal imaging. Compared with those of the control, BSA, NIR, and BF groups, the temperatures of CB- and CBF-treated M1 macrophages increased rapidly under 808-nm laser irradiation (0.96 W/cm^2^), with those of CB and CBF reaching 39.9 and 51.5 °C, respectively, after 5-min irradiation (Fig. 3J). The temperature of CB group macrophages did not reach the temperature of thermal injury. These results further confirm that CBF can target M1 macrophages and protect normal cells and tissues from damage by impairing inflammatory cell death through photothermal effects.

### In vivo therapeutic effect of CBF@LCP

**Fig. 4.**
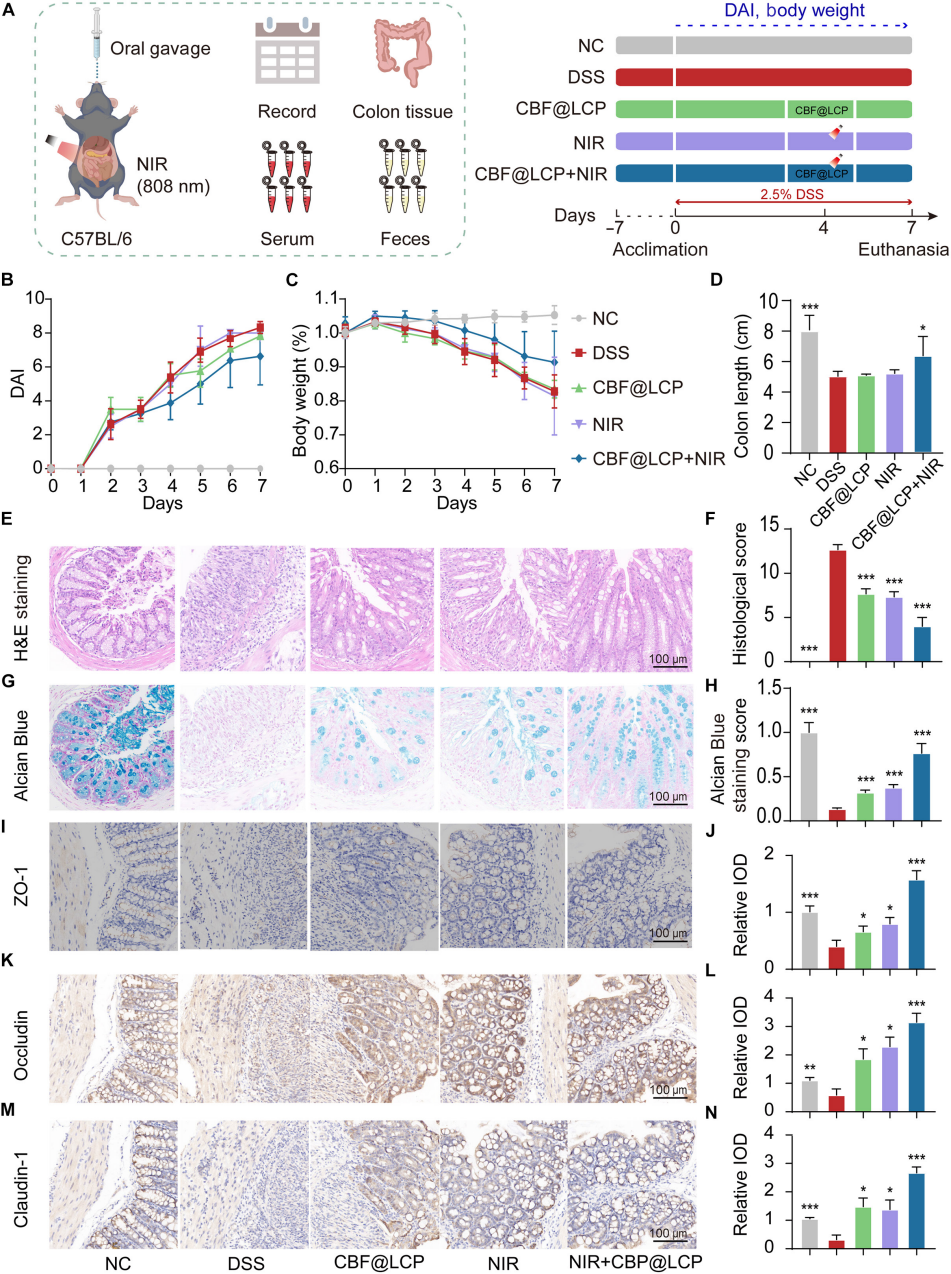
Alleviated effect of CBF@LCP in the level of phenotype and intestinal barrier. (A) Experiment design. The disease activity index (DAI) scores (B) and body weight (C) change during the animal experiment. (D) The statistical analysis for colon length in each group (*n* = 8). The colon section images and relative staining scores for hematoxylin and eosin (H&E) (E and F) and Alcian Blue (G and H) of each group. Representative image of immunohistochemical analysis and relative integrated optical density (IOD) for the tight junction (TJ) proteins of ZO-1 (I and J), occludin (K and L), and claudin-1 (M and N). Scale bar: 100 μm. *P < 0.05, **P < 0.01, and ***P < 0.001. DSS, dextran sulfate sodium.

To further investigate the treatment efficacy of CBF@LCP, we conducted a study in the DSS-induced colitis mouse model. C57BL/6 mice were randomly divided into 5 groups (*n* = 8): NC, DSS, CBF@LCP, NIR, and CBF@LCP+NIR, as outlined in the experimental protocol shown in Fig. 4A. Compared to the DSS-treated group, mice treated with CBF@LCP+NIR exhibited a significant reduction in DAI (Fig. 4B) and a notable increase in body weight (Fig. 4C), alleviating the pathological symptoms of the UC.

In the model of colitis, the length of the colon is often used as a key indicator for evaluating the degree of inflammation and pathological changes. Compared with that of the NC group, a significant reduction in colon length was observed in the DSS group. In contrast, the CBF@LCP+NIR group demonstrated the most pronounced recovery in colon length compared to the CBF@LCP and NIR groups (Fig. 4D and Fig. S7).

The intestinal epithelial barrier is an important component of intestinal health, responsible for maintaining the separation between substances in the intestinal lumen and the internal environment of the body [36]. To evaluate the integrity of the intestinal structure and the function of the intestinal barrier, we conducted H&E staining, Alcian Blue staining, and immunohistochemical analysis. As depicted in Fig. 4E and F, H&E staining revealed significant tissue damage in the DSS group, including crypt distortion, loss of goblet cells, and infiltration of inflammatory cells in the lamina propria and submucosa. These injuries were notably mitigated in the CBF@LCP+NIR group. Moreover, the deficient formation of a hollow mucus layer secreted by goblet cells in the DSS group was substantially attenuated in the CBF@LCP+NIR group, as demonstrated by Alcian Blue staining (Fig. 4G and H). At the same time, the histological score and Alcian Blue staining score of the CBF@LCP+NIR group were consistent with our observations as well. ZO-1, occludin, and claudin-1 were carefully evaluated through immunohistochemical analysis, as they are crucial proteins involved in tight junctions between epithelial cells [37]. The relative integrated optical density provided quantitative data on the expression levels of these proteins. Notably, the CBF@LCP+NIR group reversed the consequence of the reduction of their expression induced by DSS treatment and even enhanced their levels compared to that in the NC group (Fig. 4I and J). The increase in the colon length and restoration of the mucosal colon barrier provide a strong proof about the reversal of the histopathological changes in the CBF@LCP+NIR group. Overall, all of these results highlight the significant ability of CBF@LCP treatment to alleviate colitis and restore intestinal barrier function as well as enhance it.

Building upon conventional photodynamic tumor ablation strategies [38], we implemented a non-endoscopic, transcutaneous irradiation protocol to minimize invasive procedures. The existing literature supports the anti-inflammatory potential of low-dose PDT [3,8,39], leading us to hypothesize that sublethal light doses—even after cutaneous attenuation—may initiate inflammatory resolution. Consistent with this premise, amelioration of UC pathology in our in vivo models confirmed the efficacy and feasibility of this noninvasive irradiation approach.

However, certain limitations remain, including unoptimized irradiation parameters such as penetration depth and treatment frequency. Furthermore, the long-term safety profile of this strategy has yet to be systematically evaluated. Thus, additional investigations are warranted to address these aspects.

Notwithstanding these limitations, this study demonstrates the utility of an orally delivered drug system combined with external light activation as a novel PDT-based intervention for UC. This non-endoscopic strategy represents a departure from conventional intracavitary illumination approaches and may inform future translational studies aiming to develop minimally invasive PDT regimens for inflammatory diseases.

### CBF@LCP+NIR modulates inflammatory cytokine and macrophage polarization

**Fig. 5.**
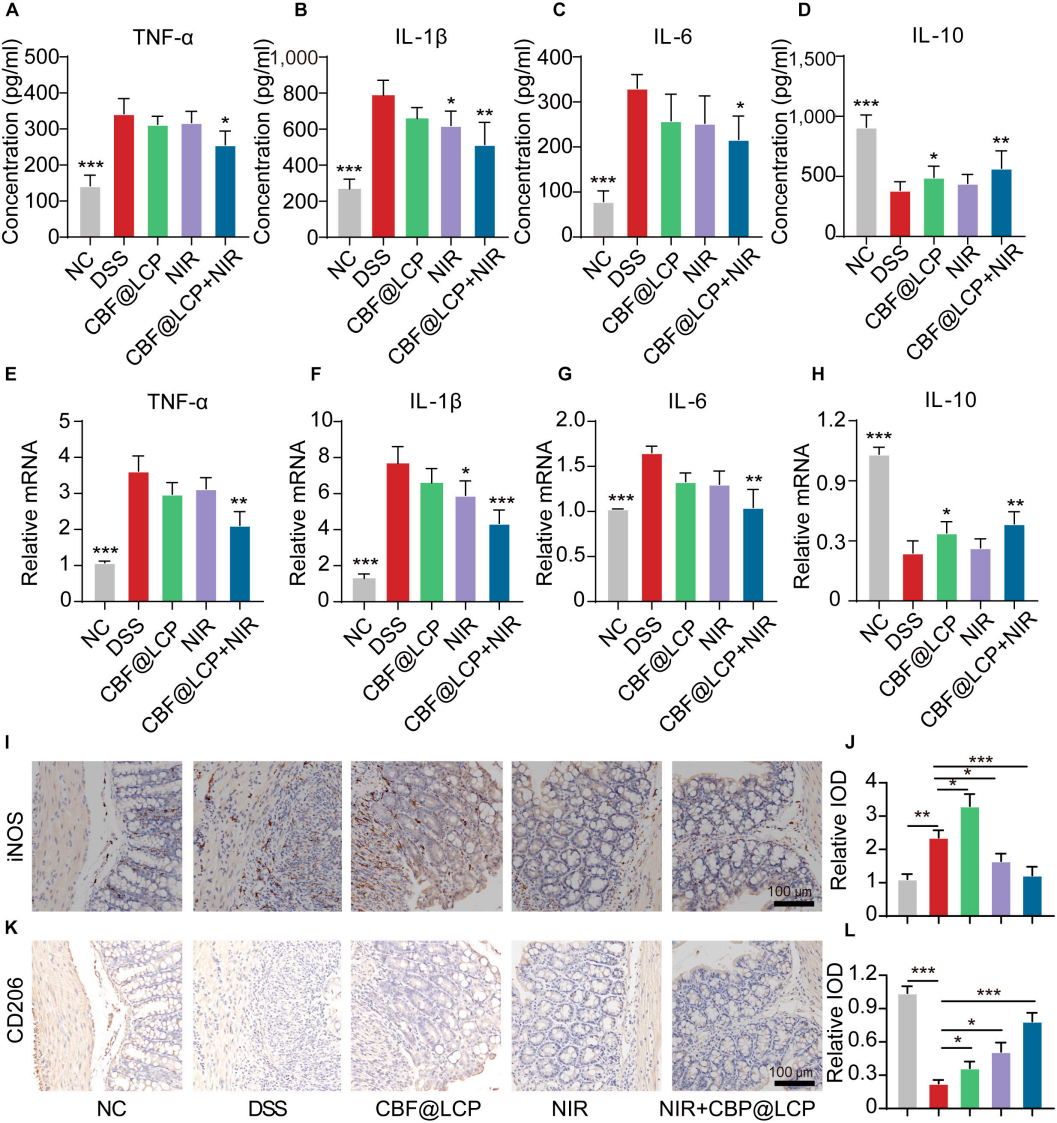
The effects of CBF@LCP+NIR in the modulation of inflammatory cytokines and macrophage polarization. The concentrations of TNF-α (A), IL-1β (B), IL-6 (C), and IL-10 (D) in serum. Relative messenger RNA (mRNA) expression of TNF-α (E), IL-1β (F), IL-6 (G), and IL-10 (H) in colon tissue. Immunohistochemical images of inducible nitric oxide synthase (iNOS) (I) and CD206 (K) expression in colon tissues. Scale bar: 100 μm. The corresponding quantitative analysis of iNOS (J) and CD206 (L) in colon tissues. *P < 0.05, **P < 0.01, and ***P < 0.001.

To verify the regulatory effects of CBF@LCP+NIR on inflammatory cytokines, the levels of inflammatory cytokines in serum (Fig. 5A to D) and colonic cytokine gene expression (Fig. 5E to H) were measured. Pro-inflammatory cytokines (TNF-α, IL-1β, and IL-6) were significantly increased in the DSS group, indicating that DSS successfully induced colitis. Notably, in the CBF@LCP+NIR treatment group, the pro-inflammatory cytokines significantly diminished, and the anti-inflammatory cytokines (IL-10) were increased. Consistently, the mRNA expression levels of TNF-α, IL-1β, and IL-6 were down-regulated, while IL-10 was up-regulated in the CBF@LCP+NIR group compared with that in the DSS group.

To further investigate whether these cytokine shifts were associated with macrophage polarization in the colon, immunohistochemical staining was performed for the M1 macrophage marker iNOS (Fig. 5I and J) and the M2 marker CD206 (Fig. 5K and L). DSS treatment significantly elevated iNOS expression, whereas CBF@LCP+NIR administration reduced iNOS expression to near-baseline levels. In contrast, CD206 expression was markedly suppressed by DSS but was significantly restored following CBF@LCP+NIR treatment. Collectively, these findings suggest that CBF@LCP+NIR alleviates DSS-induced colonic inflammation, at least in part, by modulating macrophage polarization from a pro-inflammatory M1 phenotype toward an anti-inflammatory M2 phenotype. Furthermore, combined with pathological symptom alleviation and the histopathological changes shown in Fig. 4, all that evidence gives strong proof that our therapy strategy using CBF@LCP could be effective in vivo (Figs. 4 and 5).

Macrophages are an important component of the immune system and are involved in the body’s response to infection, injury, and inflammation [40]. In UC, the polarization state of macrophages has an important influence on the pathological process [41]. M1 macrophages participate in tissue inflammatory responses by generating various pro-inflammatory cytokines and increase significantly during the onset of UC, resulting in further damage to the intestinal mucosa and aggravation of inflammation. M2 macrophages inhibit inflammatory responses through anti-inflammatory factors and are involved in tissue repair and regeneration [42,43].

With the decoration of FA, our CBF@LCP could target M1 macrophages, which was proved in CLSM (Fig. 3A to D). Moreover, in the in vivo experiments, it was observed that CBF@LCP can promote the polarization of M2 macrophages, inhibit the activity of M1 macrophages, reduce the expression of inflammatory factors to exert anti-inflammatory effects, and help alleviate the inflammatory response of UC (Fig. 5). All of these results prove that CBF@LCP could influence the polarization of macrophages and push them toward an anti-inflammatory phenotype, opening avenues for developing therapies modulating immune responses rather than simply suppressing them.

### Regulatory effect of CBF@LCP+NIR on gene expression in intestinal tissue

**Fig. 6.**
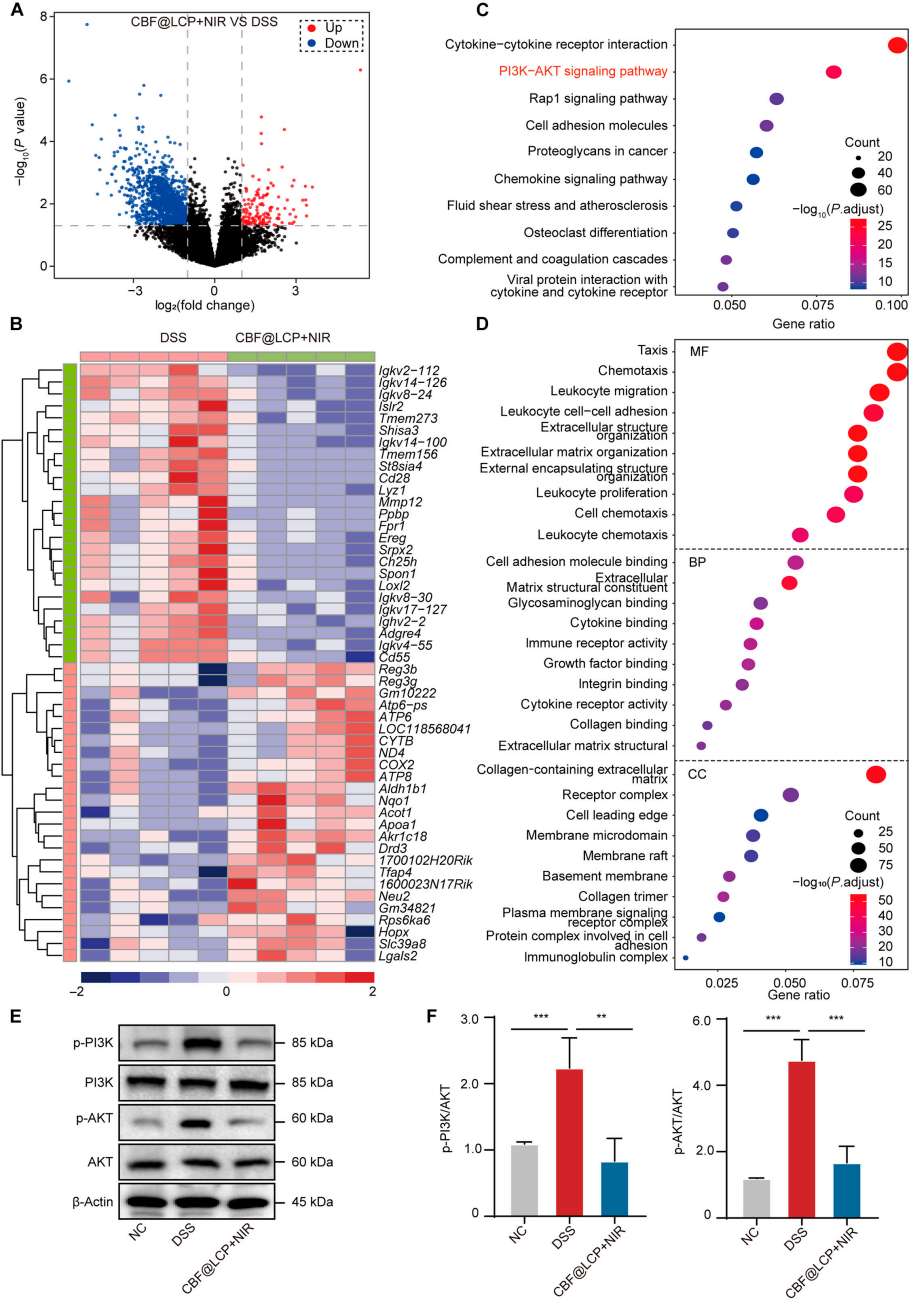
Transcriptome analysis of colitis mice treated with CBF@LCP+NIR. (A) Volcano map of differentially expressed genes (DEGs) between CBF@LCP and DSS; red represents up-regulated genes, and blue represents down-regulated genes. (B) The expression profiles of the top 50 DEGs. (C) Kyoto Encyclopedia of Genes and Genomes (KEGG) pathway enrichment analysis of the top 10 immune-related pathways. (D) The top 30 significantly enriched terms from the Gene Ontology (GO) analysis. (E) The western blot analysis of the PI3K/AKT pathway. (F) Quantitative analysis. Compared with the indicated group: **P < 0.01 and ***P < 0.001. MF, molecular function; BP, biological process; CC, cell component.

To investigate the mechanism about how combined treatment with NIR and CBF@LCP alleviates the symptoms of UC, we performed transcriptome analysis. As shown in Fig. 6A, a total of 1,437 DEGs were obtained, including 142 up-regulated and 1,295 down-regulated genes. The 50 representative DEGs are represented by a heat map (Fig. 6B). The above results show that combined treatment with NIR and CBF@LCP significantly reversed the gene expression pattern of mice with DSS-induced colitis. Kyoto Encyclopedia of Genes and Genomes (KEGG) and Gene Ontology (GO) enrichment analyses of DEGs were performed as well to better understand the underlying molecular mechanism and function of combined treatment with NIR and CBF@LCP. The KEGG pathway analysis of the top 10 immune-related pathways showed that DEGs were involved in pathways such as cytokine–cytokine receptor interaction, the PI3K/AKT signaling pathway, the Rap1 signaling pathway, cell adhesion molecules, proteoglycans in cancer, and the chemokine signaling pathway (Fig. 6C). GO enrichment was processed with 3 separate parts that include molecular function, biological process (BP), and cell component, of which BP showed significant difference after CBF@LCP administration in the DSS-induced colitis mice, which were focused on chemotaxis, leukocyte migration, leukocyte cell–cell adhesion, and extracellular structure organization (Fig. 6D). To further verify the effect of CBF@LCP combined with NIR on the PI3K/AKT pathway, we conducted western blot analysis (Fig. 6E and F). The results showed that the CBF@LCP+NIR therapy significantly down-regulated the phosphorylation levels of PI3K and AKT, indicating that CBF@LCP+NIR could effectively inhibit the activation of the PI3K/AKT signaling pathway. This finding further supports the role of CBF@LCP+NIR in regulating inflammatory responses and macrophage polarization by modulating the PI3K/AKT signaling pathway.

### CBF@LCP+NIR reshapes the relative abundance of intestinal microbiota

**Fig. 7.**
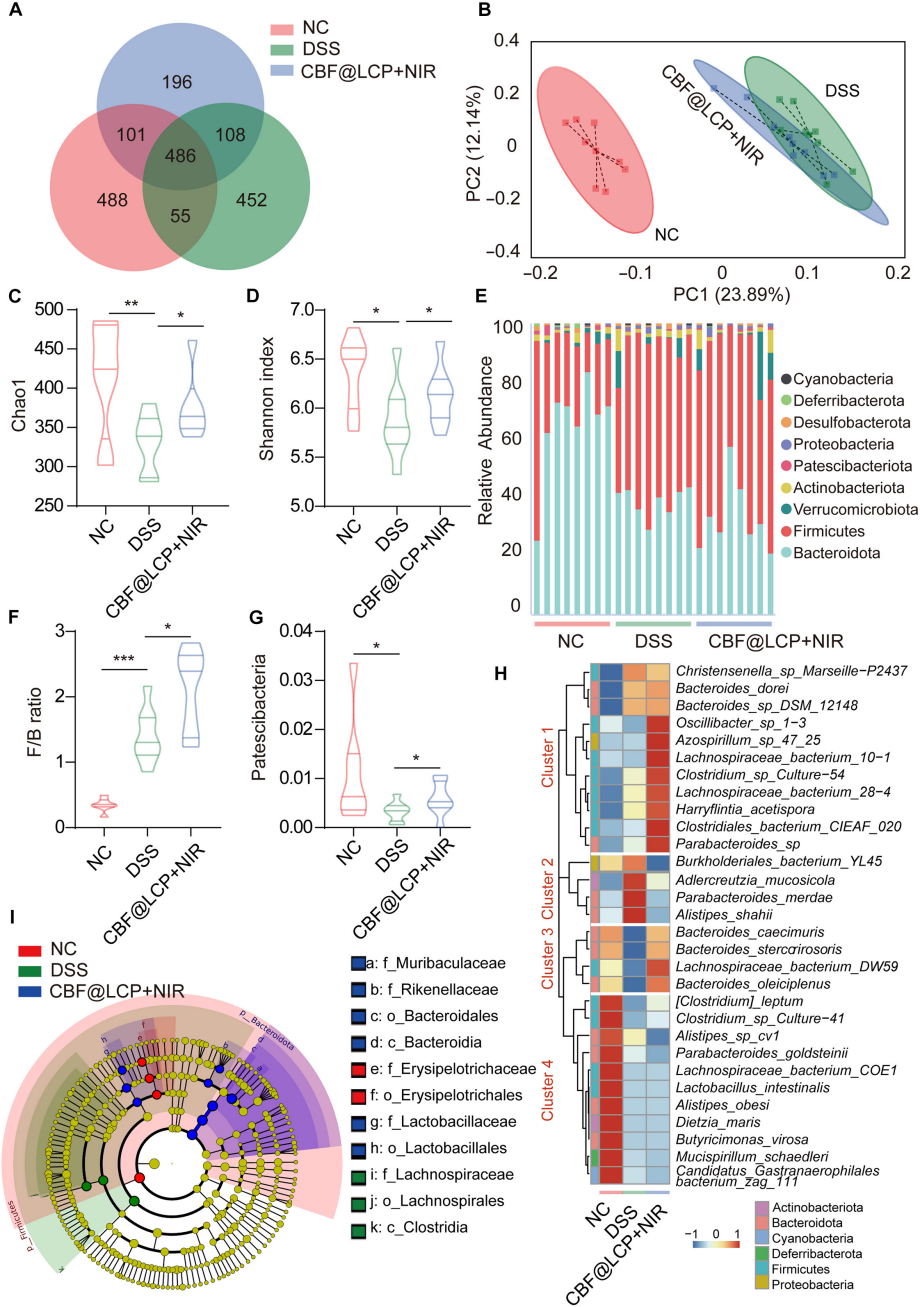
CBF@LCP+NIR modulated the overall structure of the gut microbiota. (A) The operational taxonomic unit (OTU) Venn diagram based on 16S ribosomal RNA (rRNA) sequencing. (B) Weighted UniFrac principal coordinates analysis (PCoA). (C and D) Changes in Chao1 (C) and Shannon index (D) alpha diversity. (E) The composition of the gut microbiota at the phylum level. The relative abundance of Firmicutes/Bacteroidetes (F/B) ratio (F) and Patescibacteriota (G) in 3 groups (NC, DSS, and CBF@LCP+NIR). (H) Heat map showing the relative abundance of dominant microbiome species across the NC, DSS, and CBF@LCP+NIR groups. (I) The cladogram, annotated using molecular operational taxonomic units (mOTUs). *P* < 0.05, *P < 0.05, **P < 0.01, and ***P < 0.001.

Gut microbiota has already been proved to be critically associated with UC [12]. Disordered gut microbiota could result in a pathogenic status of the colon. After induction by DSS, the UC model mouse was developed, and its composition and the diversity of the gut microbiota changed as well with an increase in harmful bacteria and a decrease in beneficial bacteria [44]. However, our therapy strategy could reverse that trend, restoring the gut microbiota (Fig. 7).

Since the restoration of gut microbiota is closely linked to mucosal recovery in UC and represents a critical biomarker for monitoring patient recovery [45], we investigated the effects of CBF@LCP treatment on the gut microbiota. The bacterial composition in fecal samples was analyzed. The common and unique operational taxonomic units (OTUs) are shown in Fig. 7A. The Venn diagram displays that 1,886 OTUs were obtained from all treatment groups, and the number of OTUs shared between the NC and CBF@LCP+NIR groups was 101, larger than the number 55 between the NC and DSS groups, indicating the shift of the species richness of gut microbiota. Using weighted UniFrac principal coordinates analysis, the microbiota composition in each treatment group was clearly grouped (Fig. 7B). The colony structure after DSS induction (blue circle) had a large distance to the NC group in the *X* axis (PC1), and the administration of CBF@LCP (green circle) changed this trend to some extent, which got closer to the NC group (red circle). After CBF@LCP+NIR treatment, there was a rebound in Chao1 (Fig. 7C) and Shannon index (Fig. 7D), demonstrating the repair of the gut microbiota. At the same time, the relative abundances of gut bacteria were compared on a phylum-by-phylum basis. As shown in Fig. 7E, the treatment of CBF@LCP activated by NIR partly reversed the DSS-induced alterations in bacterial composition. Intriguingly, the percentage of the ratio of Firmicutes/Bacteroidetes, a well-established marker of beneficial microbial restoration, was increased in the CBF@LCP+NIR group (Fig. 7F) and the percentage of Patescibacteriota was obviously increased compared with that in the DSS group (Fig. 7G), which has been reported recently to possibly have a protective effect on humans [46].

The composition of the gut microbiota was analyzed at the species level (Fig. 7H). In the DSS group, cluster 3 was reduced significantly, and in the CBF@LCP group, the decrease in cluster 3 was restored eventually, of which *Bacteroides_caecimuris* and *Bacteroides_stercorirosoris* are beneficial bacteria. In cluster 2, *Burkholderiales_bacterium_YL45*, classified as a harmful bacterium, was increased after DSS treatment, which was reversed by treatment with CBF@LCP+NIR as well. Notably, the abundance of cluster 1 was significantly higher in the CBF@LCP+NIR group than in the NC and DSS groups. Furthermore, related studies have shown that some bacteria in cluster 1 are beneficial, which indicates a kind of potential benefit of CBF@LCP+NIR treatment. The compositions of the gut microbiota at the genus and family levels are shown in Fig. S8. Using linear discriminant analysis and effect size measurements, we identified 17 bacterial taxa. The species-level cladogram annotated by molecular OTUs is shown in Fig. 7I, highlighting differentially abundant taxa among the NC, DSS, and CBF@LCP+NIR groups. Notably, the DSS group exhibited increased abundance of Clostridia (class), Lachnospirales (order), and Lachnospiraceae (family). Conversely, the CBF@LCP+NIR group showed higher levels of Lactobacillaceae, Muribaculaceae, and Rikenellaceae (family); Bacteroidia (class); and Lactobacillales and Bacteroidales (order). Linear discriminant analysis effect size analysis revealed significant differences in fecal bacterial composition among the 3 groups. CBF@LCP contains Csn and Pcn, which have prebiotic functionalities; our microbiome analysis results also indicated that combined treatment with NIR and CBF@LCP can modulate the composition of the gut microbiota.

The recovery of the intestine mucosa barrier could serve as one of the reasonable explanations for the restoration of the gut microbiota. The mucosal barrier system is associated with maintaining gut symbiosis to prevent intestinal inflammation; so when the intestinal epithelial barrier function is damaged, the prevention and suppression effect toward harmful bacteria produced by healthy mucosa cells is ruined, leading to an increase in intestinal permeability and the disorder of the gut microbiota [47]. Fortunately, related results demonstrate that our CBF@LCP could enhance the intestinal epithelial barrier (Fig. 4F to O) and improve the composition of the gut microbiota (Fig. 7), which is consistent with our hypothesis. This recovery alteration is guaranteed greatly by the probiotic coating, namely, Csn and Pcn, that with the probiotic coating, our CBF@LCP could resist erosion by gastric acid, maintain structural stability in the stomach and small intestine tract, and then could loosen its structure and be degraded by relevant enzymes from gut microbes (Fig. 2K and L), ensuring that the therapeutic effects target the colon and that CBF@LCP acts as a prebiotic to benefit colon barrier restoration and gut microbiota modulation.

Nevertheless, the way to influence the gut microbiota after the administration of CBF@LCP activated by NIR of 808 nm is unknown. Diseased colon tissue is characterized by bleeding ulcers and an increasing blood velocity due to inflammation, which leads to a higher-oxygen microenvironment compared with healthy tissue, which may be an important target for reconstructing the gut microbiota in a future study [48]. As CyI is an oxygen-dependent photosensitizer, we hypothesize that our therapy strategy toward UC could alter the gut microbiota by decreasing the oxygen concentration in the diseased colon tissue. Additionally, explanations from the perspective of gut metabolism could also offer a valid approach to understanding this change. Nevertheless, the above conjecture has no supporting data and more research is needed in the future.

Moreover, murine and human gut ecosystems differ substantially in microbial composition, metabolic function, and host immune interactions [49], which may limit direct translation of microbiota-targeted interventions. Nevertheless, our mouse model provides valuable mechanistic insight: the observation of photodynamic remodeling of the gut microbiota and the resulting macrophage polarization validates the core therapeutic principle of our approach. Thus, despite inherent species differences, these results offer proof-of-concept mechanistic validation of microbiota modulation as an effective anti-inflammatory strategy.

### Integrative omics reveals the mechanisms by which CBF@LCP exerts anticolitis effects

**Fig. 8.**
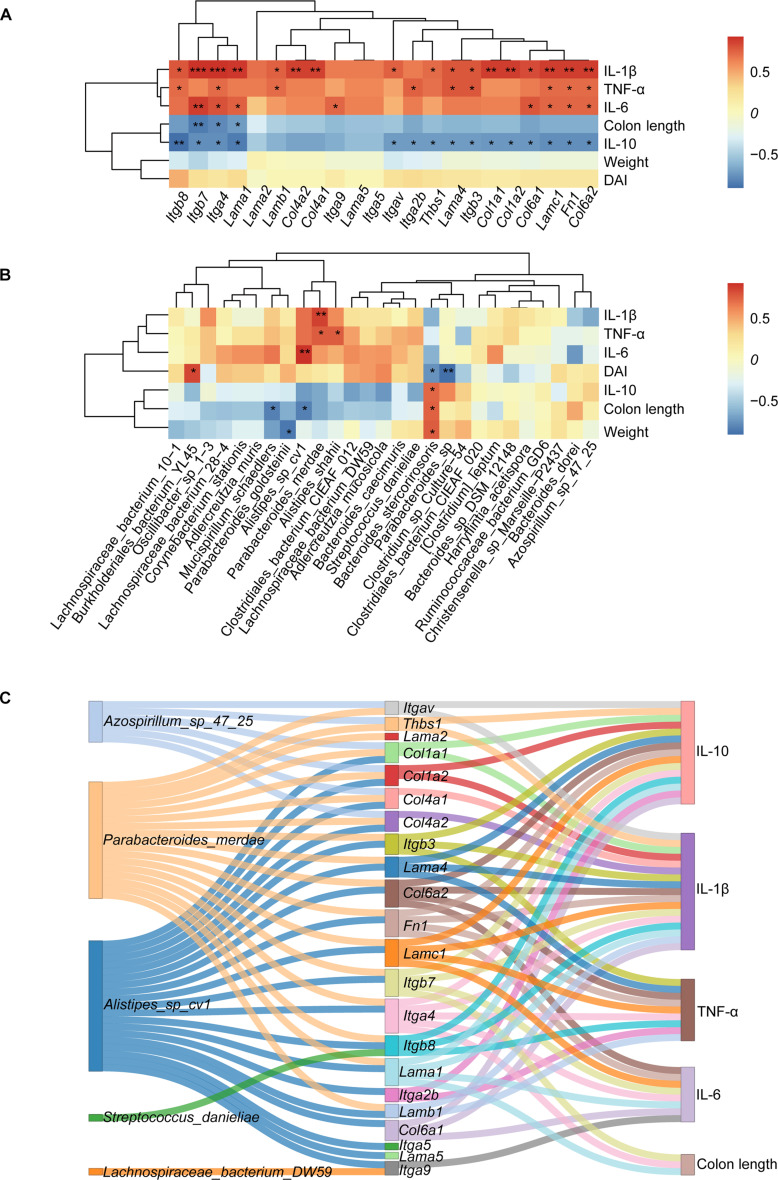
Integrated multi-omics analyses reveal interconnected potential mechanisms of CBF@LCP. (A) The Spearman correlation heat map between genes associated with the PI3K/AKT signaling pathway and phenotypic indicators. (B) Correlation heat map of differentially abundant bacterial taxa and phenotypic indicators. (C) Sankey plot illustrating the interactions between gut bacteria, genes, and phenotypic indicators. *P < 0.05, **P < 0.01, and ***P < 0.001.

To elucidate the underlying mechanisms of combined treatment with NIR and CBF@LCP in ameliorating UC, we performed an integrative multi-omics analysis, combining transcriptomics, microbial data, and phenotype indicators. Spearman correlation analysis was employed to investigate the relationships between DEGs associated with the PI3K/AKT signaling pathway and phenotypic indicators. As shown in Fig. 8A, DEGs such as *Itga4*, *Lama1*, *Fn1*, and *Col6a2* exhibited significant positive correlations with the pro-inflammatory cytokines IL-6, TNF-α, and IL-1β (*P* < 0.05) but significant negative correlations with the anti-inflammatory cytokine IL-10 and colon length (*P* < 0.05). These findings suggest that CBF@LCP combined with NIR mitigates UC by modulating the PI3K/AKT signaling pathway, thereby suppressing inflammatory responses and improving phenotypic outcomes. Similarly, Spearman correlation analysis was conducted to explore associations between microbial taxa and phenotypic indicators. As depicted in Fig. 8B, *Bacteroides stercorirosoris* was significantly positively correlated with body weight, colon length, and IL-10 levels, while it was negatively correlated with DAI, IL-6, IL-1β, and TNF-α (*P* < 0.05). In contrast, *Alistipes shahii*, *Parabacteroides merdae*, *Alistipes* sp. *cv1*, and *Burkholderiales bacterium YL45* showed significant positive correlations with the pro-inflammatory cytokines IL-1β, IL-6, and TNF-α (*P* < 0.05), indicating their potential role in exacerbating inflammation.

To further clarify the mechanistic interplay between microbial taxa, host gene expression, and phenotypic outcomes, a Sankey plot was constructed based on Spearman correlations (|*r*| > 0.6), integrating 5 microbial taxa, 22 DEGs related to the PI3K/AKT pathway, and 5 phenotypic indicators (Fig. 8C). Notably, *P. merdae* and *Alistipes* sp. *cv1* exhibited strong correlations with the majority of PI3K/AKT-related DEGs, which in turn were closely associated with phenotypic indicators. In particular, *Alistipes* sp. *cv1* and *P. merdae* are significantly reduced in the CBF@LCP-treated group (Fig. 7I), and *Parabacteroides* has been implicated in promoting inflammation in certain contexts [50], which could serve as a potential target for further future studies.

Given the documented involvement of the PI3K/AKT pathway in enteritis and its known role in promoting inflammation and impairing mucosal healing, our results align with prior evidence that its suppression enhances recovery in inflammatory bowel disease models [25]. Consistently, we observed down-regulation of PI3K/AKT signaling following CBF@LCP+NIR treatment, supporting its contribution to therapeutic efficacy.

Collectively, our findings indicate that CBF@LCP alleviates UC through a multifaceted mechanism. First, the prebiotic shell contributes to the remodeling of gut microbiota, promoting beneficial bacteria while suppressing harmful taxa. In parallel, the nanoplatform targets M1 macrophages and modulates their polarization by down-regulating the PI3K/AKT signaling pathway, thereby dampening inflammatory responses. Additionally, CyI-induced photodynamic activation consumes local oxygen, creating a more favorable environment for anaerobic microbes. This microbiota shift, together with improved immune regulation, leads to enhanced intestinal barrier integrity and reduced mucosal inflammation. These interconnected effects ultimately create a reinforcing therapeutic loop that restores immune and microbial homeostasis in the colonic environment.

Therefore, we propose that CBF@LCP exerts its therapeutic action by targeting M1 macrophages to suppress PI3K/AKT signaling, thereby reducing inflammation and restoring mucosal integrity. This recovery, in turn, modulates pro-inflammatory microbial taxa, including *Alistipes* sp. *cv1* and *P. merdae*, reinforcing a self-sustaining anti-inflammatory loop.

## Conclusion

Overall, our colon-targeted oral Csn/Pcn-coated photosensitizer NPs CBF@LCP was successfully constructed and its photodynamic therapeutic efficacy was validated in a DSS-induced UC mouse model. This was the first study of PDT for treating colitis through oral administration, and we initiatively made an overview of how to design experiments to verify the feasibility and effectiveness of PDT toward UC via oral delivery, which could contribute a lot to the improvement of the efficiency of subsequent research. The results both in vitro and in vivo provide strong evidence that our design of a photosensitizer is effective, applicable, and promising for relieving colitis, which would encourage more research into diverse and enhanced oral delivery systems for PDT in the future. Through integrated multi-omics analyses, we deduce that the potential mechanism of colitis alleviation may be the alteration of the composition and diversity of gut microbiota and the inhibition of the PI3K/AKT signaling pathway. Nevertheless, more study is needed to elucidate the comprehensive mechanism from the perspective of genes and transcription. In the future, we plan to conduct relevant explorations at the metabolic and immune levels to improve our understanding of the study. Meanwhile, regarding the speculative mechanism by which oxygen consumption shifts the gut microenvironment, we will further verify this conjecture through some methods like oxygen quantification and oxygen monitoring.

## Ethical Approval

Animal protocols were reviewed and approved by the Ethics Committee of the Medical College of Qingdao University (QDU-AEC-2023388).

## Data Availability

The authors confirm that the data supporting the findings of this study are available at PRJNA1203181 (NCBI). The data supporting this article are found within the text and the Supplementary Materials file. Any additional data and the data that support the plots within this paper are available from the corresponding authors upon reasonable request.
