# Supplementary material for: Prebiotic-Engineered Oral Nanoplatform against Ulcerative Colitis via Photodynamic Remodeling of Gut Microbiota and Macrophage Polarization
Source: Biomater Res. 2025 Nov 21;29:0283. doi: 10.34133/bmr.0283 (PMC12635410; doi:10.34133/bmr.0283)
Supplement: Supplementary 1 — Table S1 Figs. S1 to S8 [file bmr.0283.f1.docx]

**Prebiotic-engineered Oral Nanoplatform against Ulcerative Colitis *via* Photodynamic Remodeling of Gut Microbiota and Macrophage Polarization**

Ningning He ^1, 2, #^, Huimei jiang ^3, #^, Tong Dai ^2, #^, Geun-soo Kim^2^, Peng Liu^2^, Yifan Zhao^3^, Shangyong Li ^2,^ *, Jie Cao^3,^ *, Zequn Li^1, 4,^ *

*Supplementary Material*

**Table S1** Gene name and primers sequence

| **Gene name** | **Primers sequences** |
| --- | --- |
| TNF-α | Forward: 5’-AACTCCAGGCGGTGCCTATG-3’ |
|  | Reverse: 5’-TCCAGCTGCTCCTCCACTTG-3’ |
| IL-1β | Forward: 5’-TCGCAGCAGCACATCAACAAGAG-3’ |
|  | Reverse: 5’-AGGTCCACGGGAAAGACACAGG-3’ |
| IL-6 | Forward: 5’-AAGTCCGGAGAGGAGACTTC-3’ |
|  | Reverse: 5’-TGGATGGTCTTGGTCCTTAG-3’ |
| IL-10 | Forward: 5’-TTCTTTCAAACAAAGGACCAGC-3’ |
|  | Reverse: 5’-GCAACCCAAGTAACCCTTAAAG-3’ |
| GAPDH | Forward: 5’-TGGAGAAACCTGCCAAGTATGA-3’ |
|  | Reverse: 5’-TGGAAGAATGGGAGTTGCTGT-3’ |


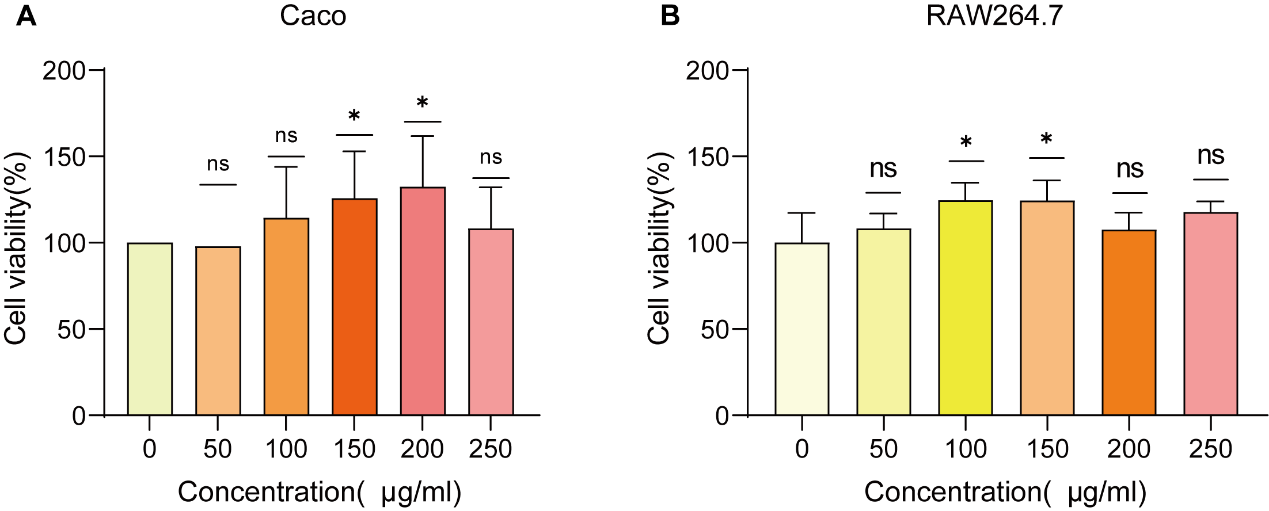


**Fig. S1.** The cell viability for Caco cells and RAW264.7. *p < 0. 1


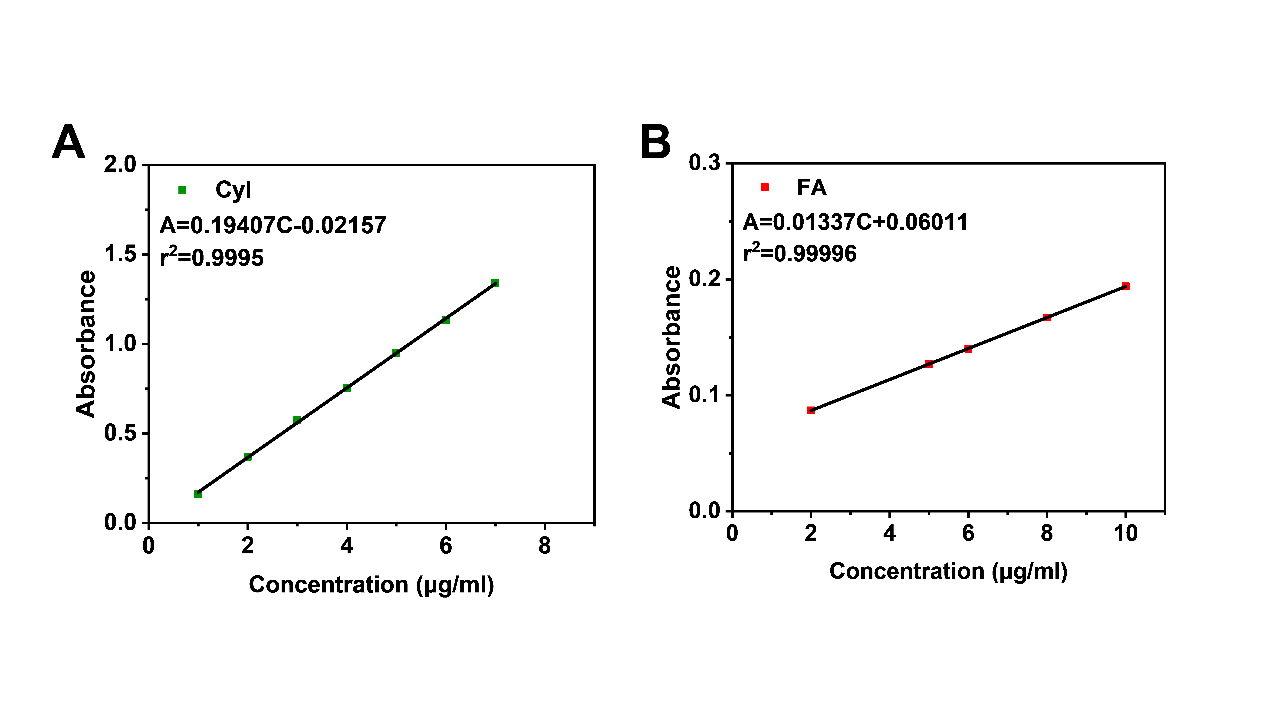


**Fig. S2.** The standard curve for CyI (A) and FA (B)


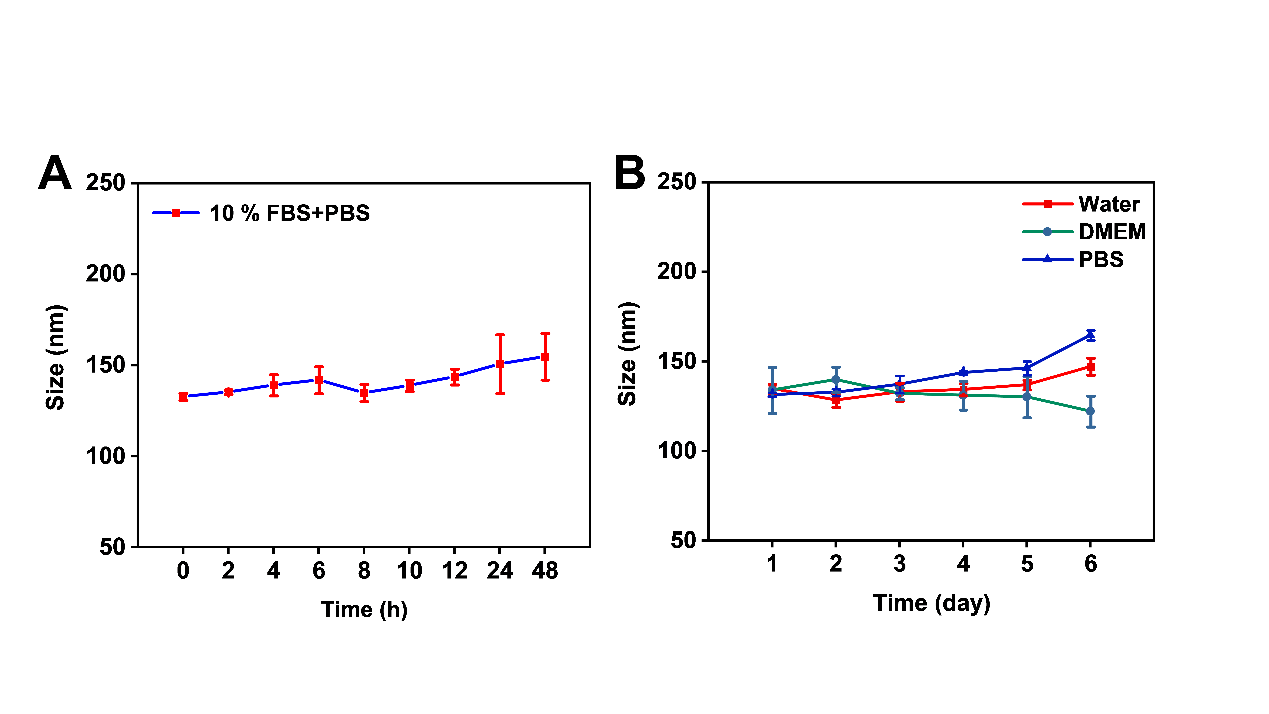


**Fig. S3.** Stability of the CBF@L. (A) Serum stability of CBF@L in PBS containing 10 % FBS at 37°C (n=3); (B) Stability of CBF@L placed in different systems at 4°C (n=3).





**Fig. S4.** Temperature change curves of BSA, FA, CBF, CBF@L and CBF@LCP upon laser irradiation (0.96W/cm^2^, 808 nm, 5 min) (n=3).

**Fig. S5.** Release curves of CBF@LCP with a bacterial solution or pectinase in the simulated digestive system.

**Fig. S6.** Mean fluorescence intensity of M1 macrophages with different treatments, data are presented as means ± SD (n=3), ***p < 0.001.


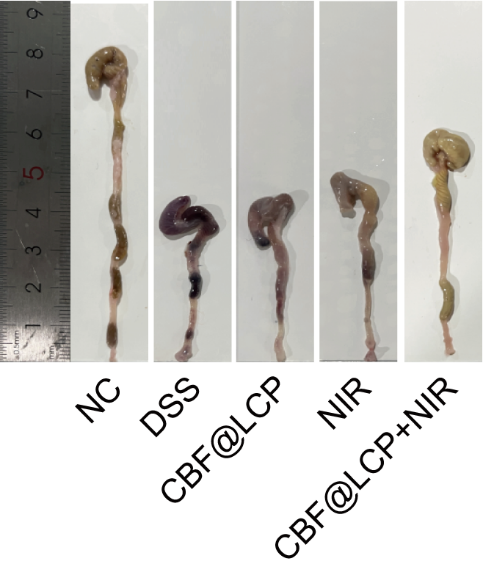


**Fig. S7.** The dissected colon tissue image of each group.


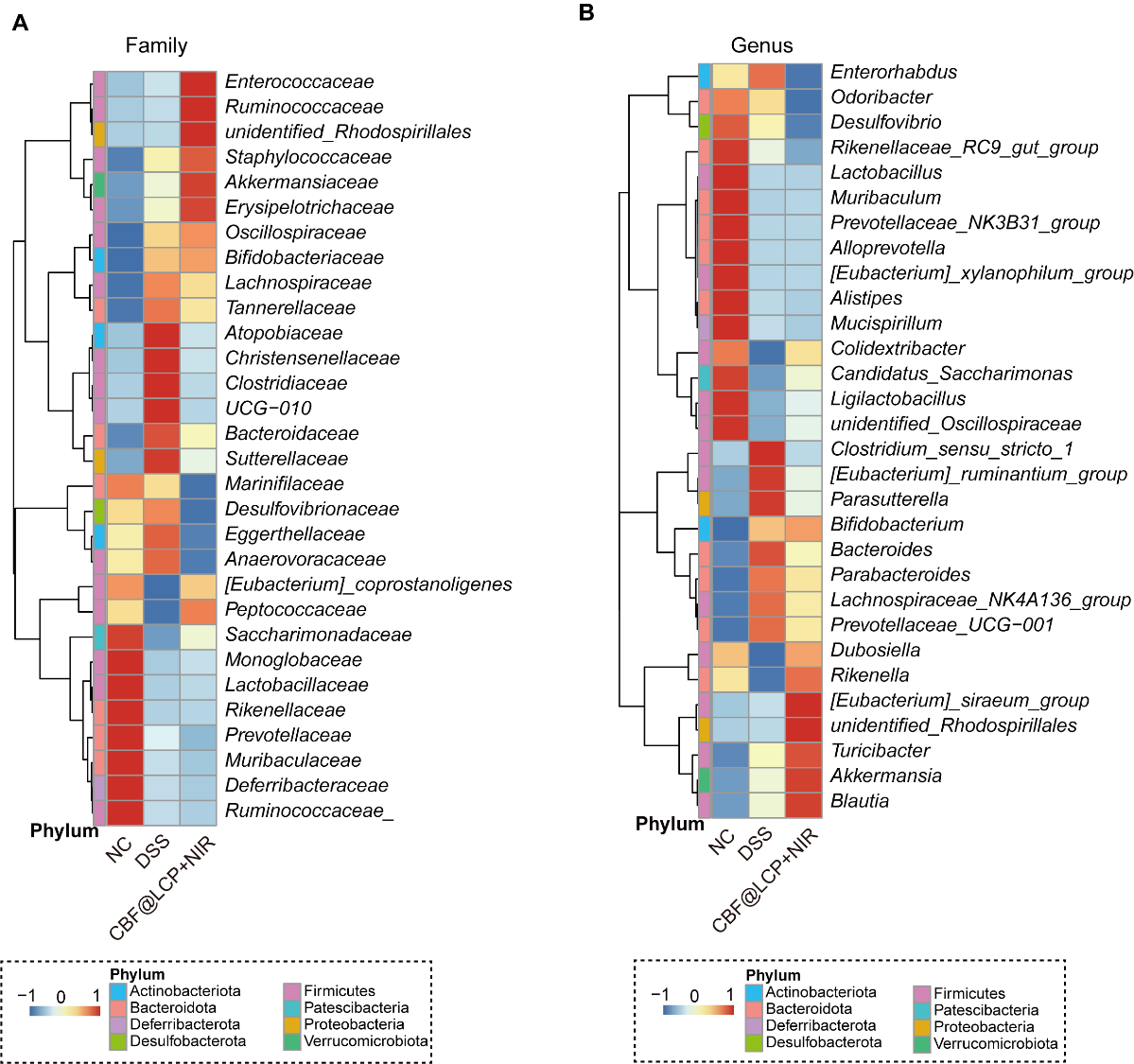


**Fig. S8.** The composition of the gut microbiota at the family (A), genus (B) levels.
